# Resilience and Stress as Mediators in the Relationship of Mindfulness and Happiness

**DOI:** 10.3389/fpsyg.2022.771263

**Published:** 2022-02-03

**Authors:** Badri Bajaj, Bassam Khoury, Santoshi Sengupta

**Affiliations:** ^1^Humanities and Social Sciences, Jaypee Institute of Information Technology, Noida, India; ^2^Department of Educational & Counselling Psychology, McGill University, Montreal, QC, Canada; ^3^School of Management, Graphic Era Hill University, Bhimtal, India

**Keywords:** mindfulness, resilience, stress, happiness, AMOS

## Abstract

The aim of the present study was to examine the mediation effects of resilience and stress, two perceived opposite constructs, in the relationship between mindfulness and happiness. Mindful Attention Awareness Scale, Connor–Davidson Resilience Scale, Subjective Happiness Scale, Depression Anxiety Stress Scales short version-21 were administered to 523 undergraduate university students in India. Structural Equation Modeling with bootstrapping was applied to test the mediating effects of resilience and stress. Results showed that resilience and stress partially mediated the mindfulness-happiness relationship. In addition, resilience partially mediated the relationship of mindfulness to stress. Findings suggest that mindfulness may play an influential role in enhancing happiness through the mediating effects of resilience and stress.

## Introduction

Happiness is unquestionably a personal investment worthy of pursuit (Howells et al., 2016; Srivastava and Muhammad, 2021), which is believed to create life successes and also authenticate existence (Lyubomksky et al., 2005). Most of the human race attempts to achieve a state of thriving by rating themselves as satisfied with lives and being happy (Myers, 2000). As a direct indicator of quality of life, happiness is an invaluable personal goal pursued by all individuals (Tay et al., 2015; Lord et al., 2020) as it has positive effects on longevity and on many positive life outcomes (Seligman et al., 2005; Lawrence et al., 2015). Individuals associate happiness with multiple personal benefits such as health, increased earnings, longer life expectancy, better social relationships, and a happier marriage (Lyubomirsky et al., 2005; Diener et al., 2017, 2018). Thus, in the contemporary times, happiness has become a trending area for academic, management and national research (Veenhoven, 2015).

Mindfulness is an English translation of the 2,500 year-old Pali word “sati,” a term which according to Theravada Buddhism connotes “awareness,” “attention” and “remembering or intention” (Bodhi, 2011; Analayo, 2013). Mindfulness can be also conceptualized as a state, which can be induced through different practices (e.g., meditation), or as a trait, which is a stable disposition (Brown and Ryan, 2003). Kabat-Zinn (1994) defined mindfulness as “the awareness that arises from paying attention on purpose, in the present moment, non-judgmentally.” Comprising of self-regulation of attention and orientation to experience, mindfulness helps in establishing continuing contact with experience and it may contribute to many factors related to psychological health (Keng et al., 2011) and happiness (Coo and Salanova, 2018).

Resilience embodies the personal qualities in individuals, which enable them to thrive in stressful and adverse situations (Connor and Davidson, 2003). Individuals can maintain their psychological and physical health as resilience provides them protective factors that help in absorbing negative outcomes during difficult times (Ryff and Singer, 2000; Connor and Davidson, 2003). Resilience also plays a vital role in avoiding negative behavioral outcomes (Ryff and Singer, 1996; Ryff et al., 1998) and in enhancing overall happiness.

Stress may comprise of both minor stressful events and major stressful life events, which bring a major change in individual’s circumstances or status (Wouters et al., 2018) and are associated with a range of psychological disorders (Francis et al., 2012; Roos et al., 2018). Psychological stress extends the risk for chronic diseases, which constitute the greatest threat to public health, including heart disease (Cohen et al., 2007). Taking into consideration the relationships between stress, physical health, and poor health behaviors; (McEwen, 2007; Sirois, 2007; Jain and Cohen, 2013), it becomes imperative to study how to decrease stress levels.

Interestingly, prior research suggests that mindfulness leads to positive affect and subjective vitality, and prevents the experience of negative affect (St-Louis et al., 2018). Specific to our study, we find that mindfulness is correlated with both resilience and stress. Subjective sense of happiness may be increased by increasing resilience (Hwang et al., 2018), and by reducing stress (Schiffrin and Nelson, 2010). Though mindfulness is conducive for happiness as it facilitates awareness of what is worth doing, and doing it well (Ryan et al., 2008) little is known about how effectively mindfulness translates into happiness by simultaneously decreasing stress and enhancing resilience. Prior research suggests individual relationships among mindfulness, stress, resilience and happiness (Tran et al., 2014; Coo and Salanova, 2018; Hwang et al., 2018); however, to the best of our knowledge, no study has investigated effects of mindfulness on happiness through both resilience and stress together as mediators of these effects. This is particularly important as most studies focusing on stress, emphasize psychopathology rather than human strengths, whereas studies focusing on happiness tend to emphasize human strengths and qualities, resilience being a central one (Snyder et al., 2011).

Addressing the need for a better conceptual understanding of the mechanism behind translating mindfulness to happiness, this paper attempts to investigate the role of resilience and stress, two perceived opposite constructs by drawing together prior academic research and a judiciously designed empirical study. We position our study on a sample of university students as resilience is viewed as an asset that supports university students’ mental health requirements (Hartley, 2012; Kelifa et al., 2021). Secondly, university students experience more stress and issues related to mental health as compared to their peers from a non-university background (Stallman, 2010; Karyotaki et al., 2020). And lastly, life at a university can be quite complex and demanding, which may require them to exercise ways of coping with stress and high pressure demands of competitive academic/coursework demands, striking balance between study and life, issues related to relationships and financial problems (Karyotaki et al., 2020). Thus, the current study may shed light on some potential psychological mechanism such as mindfulness which may help in improving university students’ well-being. Examining mindfulness, resilience, and stress in university students will further contribute to knowledge in the field of happiness.

## Theoretical Foundations and Hypotheses Development

### Self-Determination Theory

We place our study within the theoretical framework of self-determination theory (SDT) (Ryan and Deci, 2000), which is an “organismic dialectic” approach to human motivation which has received extensive empirical validation in several life domains (Ryan and Deci, 2017). Three basic psychological needs for autonomy, competence, and relatedness stand at the core of SDT, which necessitate fully functional, healthy, and wellness-filled life (Ryan, 2013). While the need for autonomy refers to behavioral experience which is out of will, chosen, and done with a reflective sense, need for competence refers to the experience of skill development and gaining mastery in behavioral pursuits. Similarly, need for relatedness refers to the experience of respect toward others who are important to an individual. All these needs are key psychological nutrients that are essential for psychological growth, integrated functioning, and well-being.

Mindfulness is aptly postulated in SDT. Typified as an “allowing” form of awareness, mindfulness quiets the ego and bares the attention to witness internal and external events as naturally as they occur. There is no defense or cognitive distortion while viewing the events, rather a more objective, dispassionate view of events (Shapiro et al., 2007) marked by a clear and reflective mind and clarity of mind (Narayanan and Moynihan, 2006). As such, individuals with higher levels of mindfulness feel lesser amounts of stress and experience lesser physical complaints. Prior literature suggests that mindfulness leads to better awareness of internal phenomena and external conditions that an individual is experiencing. Internal phenomena may include emotions and needs, and external conditions may include pressures and stress. Thus, mindful people are in a better position to engage in reflective choices and actions that are congruent with their self (Ryan and Deci, 2017). Relevant to our study, research has shown that individuals who are higher in mindfulness are less likely to experience basic psychological need frustration (Schultz et al., 2015) such as stress. Mindfulness supports self-regulation, which is a more autonomous form, and also backs more intrinsic versus extrinsic goal selection, thereby leading to a meaningful life and happiness.

### Mindfulness and Happiness

Mindfulness is inherently linked to greater happiness (Huta and Ryan, 2010). Mindfulness promotes happiness by bringing greater clarity and vividness to current experience without filtering it through any discriminatory thought (Brown and Ryan, 2003). Mindfulness stimulates an upward spiral of positive affect and cognition, which contributes to higher happiness (Garland et al., 2015). Prior research indicates that mindfulness is positively associated with happiness (Coo and Salanova, 2018; Chin et al., 2019). Considering the rationale discussed above and the empirical evidence provided, it is feasible to hypothesize that mindfulness is positively related to happiness.

### Resilience as Mediator Between Mindfulness and Happiness

Mindfulness was shown to have the potential to cultivate resilience (Nila et al., 2016; Wang et al., 2016; Hwang et al., 2018). People with high levels of mindfulness gain enhanced ability to respond appropriately to difficult situations without reacting in automatic and non-adaptive ways (Langer and Moldoveanu, 2000; Wallace and Shapiro, 2006; Sass et al., 2019). Mindfulness training is an efficacious intervention for enhancing resilience (Felver et al., 2018; Zarotti et al., 2020) and the association between the two has been confirmed in various empirical studies (Pidgeon et al., 2014; Kemper et al., 2015).

Resilience plays an important role in increasing positive psychological outcomes; and decreasing negative outcomes (Ryff and Singer, 1996; Ryff et al., 1998) and in enhancing overall happiness (Bajaj and Pande, 2016). Prior research has shown that resilience is a human strength that may have a considerable impact on subjective well-being or happiness (Lü et al., 2014; Tan et al., 2021). Resilient individuals are more persistent in adverse situations, cope better with everyday difficulties and have more capacity to respond to life stressors (Mandleco and Peery, 2000; Hays-Grudo et al., 2021). Character strengths such as hope, zest, and bravery play an instrumental role in resilience-related factors such as optimism and positive affect (Martínez-Martí and Ruch, 2017), which eventually help resilient individuals face stressors in a positive way. When faced with a stressor, such individuals experience more positive emotions and thus, are able to quickly rebound from stress (Ong et al., 2006). Therefore individuals with higher resilience can maintain physical and psychological health by absorbing negative consequences of difficult times (Connor and Davidson, 2003).

Based on the associations of mindfulness and happiness and the potential role of resilience in mindfulness and happiness, it is plausible to suggest that resilience mediates the relationship between mindfulness and happiness.

### Stress as a Mediator Between Mindfulness and Happiness

It is fascinating to see that prior research gives us evidence that mindfulness has the potential to reduce mental health issues such as stress, and enhance wellbeing-related outcomes such as happiness (Lomas et al., 2019). Negatively related with stress (Tran et al., 2014; Kriakous et al., 2021), mindfulness improves an individual’s ability to deal with life stressors (Duprey et al., 2018) by reducing the propensity to perceive situations in ways that activate stress (Shapiro et al., 2007). Higher mindfulness may lead to lowered stress due to decreased negative cognitive appraisals of threatening events and experiences. Individuals having high mindfulness levels are less likely to appraise their day-to-day experiences as stressful (Weinstein et al., 2009; Arlt Mutch et al., 2021). Their ability to regulate their emotions may make them more adaptable to various stressors in their environment as they behave in ways that are consistent with their values (Brown and Ryan, 2004; Palmer and Rodger, 2009).

It is a common conception that stress impedes happiness (Schiffrin and Nelson, 2010). Chronic stressors may significantly affect the development of negative emotional responses which may lead to lower happiness (Schiffrin and Nelson, 2010; Eppelmann et al., 2016) suggesting that individuals with less stress levels may experience increased levels of happiness. Ruiz-Aranda et al. (2014) in their longitudinal research found that individuals with high emotional intelligence evaluated situations as less stressful, which resulted in higher happiness. Managing stress may lead to reducing worrisome habits, which may further lead to increase in happiness (Kaiser-Greenland, 2010).

Given the associations between mindfulness, stress, and happiness, we can suggest that mindfulness plays an important role in decreasing stress and stress-related outcomes and increasing subjective sense of happiness and its related outcomes. Considering how mindfulness is instrumental in decreasing stress and how lowered levels of stress may increase happiness, we hypothesize that stress may mediate the relationship of mindfulness with happiness.

### Resilience to Stress

Resilience is a stress resistance resource for individuals who experience traumatic life circumstances as it buffers the negative impact of stress. The concept of resilience explains why some individuals, in spite of high levels of stress, thrive and get enhanced levels of ability to manage future challenges (Kinman and Grant, 2011; Komachi and Kamibeppu, 2018). In a review of resilience and stress, Ozbay et al. (2007), suggested that resilience has the potential to reduce the effects of stress. Recent studies have suggested that enhanced resilience may help in mitigating the negative effects of occupational stressors (Hao et al., 2015; Kaplan et al., 2017). Thus, we hypothesize that resilience is negative related to stress.

### Resilience as Moderator Between Mindfulness and Happiness

Previous studies have also shown that resilient individuals could maintain their physical and psychological health both through buffering negative consequences from difficult times and through improving psychological well-being (Ryff and Singer, 2000; Connor and Davidson, 2003). Resilience is also thought to be an important protective factor against the development of psychiatric disorder in the face of adversity (Rutter, 1985). From above reasoning, it was hypothesized that resilience may also act as a moderator between the relationship of mindfulness to happiness. Thus, we hypothesize that resilience moderates the relationship between mindfulness and happiness.

## Materials and Methods

### Participants and Procedure

We invited students from an Indian university to participate in our study. 523 undergraduate students (359 males and 164 females) were recruited based on their consent to participate in the research. These students were pursuing undergraduate degree in engineering. Their average age was 20.1 years (SD = 1.3), wherein highest age was 24 and lowest age was 17. After obtaining informed consent, participants were asked to complete scales of mindfulness, resilience, stress, and happiness, in classroom.

### Measures

#### Mindfulness

The 15-item Mindful Attention Awareness Scale (MAAS) was used to measure participants’ mindfulness scores (Brown and Ryan, 2003). The MAAS is a 15 items scale. It is a six-point rating scale that ranges from “almost always,” to “almost never.” An example item is: “I rush through activities without being really attentive to them.” The MASS has demonstrated good internal consistency, and good convergent and discriminant (Brown and Ryan, 2003) validity. The internal consistency of the present sample was 0.82.

#### Resilience

The Connor–Davidson Resilience Scale (CD-RISC) was used to measure participants’ resilience scores (A10-item version of The CD-RISC was administered) (Campbell-Sills and Stein, 2007). An example item is: “See humorous side of problems.” This scale has demonstrated good internal consistency and construct validity in prior research (Campbell-Sills and Stein, 2007). The internal consistency of the present sample was 0.84.

#### Happiness

The Subjective Happiness Scale (SHS) was used to measure participants’ happiness scores. The SHS is a four-item scale that assesses participants’ subjective sense of global happiness. This scale is a seven-point scale (e.g., from less happy to more happy). An example item is “Compared to my peers, I consider myself…” This scale has demonstrated good levels of reliability and validity (Lyubomirsky and Lepper, 1999). The internal consistency of the present sample was 0.80.

#### Stress

Stress was assessed using the short version of Depression Anxiety Stress Scales (Lovibond and Lovibond, 1995). Seven items for stress were adapted from DASS-21 to assess stress level of individuals. The participants rated themselves on a Likert scale that ranged from 0 (did not apply to me) to 3 (applied to me very much or most of the time). It includes items such as, “I tended to over-react to situations.” This scale has also demonstrated good levels of reliability and validity (Hamill et al., 2015). The internal consistency of the present sample was 0.72.

### Data Analysis

Means, standard deviations, and correlations of the variables were computed using SPSS 22.0. The role of resilience and stress as mediators was tested via structural equation modeling (SEM), which is a multivariate technique used to analyze observed and latent variables relationships. It can be viewed as a combination of factor analysis and multivariate regression analysis. Two methods are widely used in power analysis for SEM. One is based on the likelihood ratio test proposed by Satorra and Saris (1985). The other is based on RMSEA proposed by MacCallum et al. (1996). This function is for SEM power analysis based on RMSEA.

We followed the two-step procedure of SEM using AMOS 18.0. First, we calculated the measurement model, and satisfactory results were obtained. Then, in the second step, we examined the structural model. We used indices recommended by Hu and Bentler (1999) and Kline (2011) to assess the overall fit of the model to data. We divided the items of each latent factor into parcels to control inflated measurement errors. A random assignment approach was used to create parcels (Little et al., 2002). Three parcels for mindfulness latent factor and resilience latent factor and stress latent factor were formed. Happiness latent factor was defined using all items of the SHS as it consisted of only four items.

## Results

**FIGURE 1:**
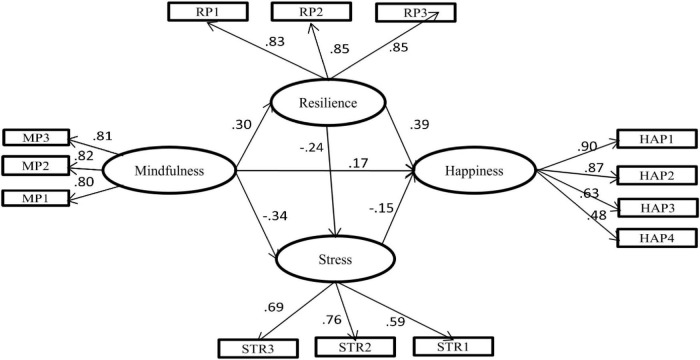
The Structural Equation Model regarding the mediating effect of resilience in the relationship of mindfulness to happiness, and stress. Factor loadings are standardized. MP1–MP3 = three parcels of mindfulness; RP1–RP3 = three parcels of resilience; STR1-STR3 = three parcels of stress.

Means, standard deviations, and correlations of all the study variables are shown in Table 1. The measurement model of the study comprised 13 observed variables and four latent variables (mindfulness, resilience, stress, and happiness). This model showed an excellent fit to the data: χ2 = 81.9; df = 59; χ2/df = 1.39; RMSEA = 0.027; PClose = 0.999; SRMR = 0.031; and CFI = 0.992. All indicators truly represented their latent factors. Then, we tested the structural model. A partially mediated model, Model 1, (with resilience and stress as mediators) and two direct paths one from resilience to happiness and one from stress to happiness revealed an excellent fit to the data: χ2 = 100.56, df = 60, χ2/df = 1.68; CFI = 0.986; SRMR = 0.051; RMSEA = 0.036; and PClose = 0.974. The results showed that the direct path coefficients in the proposed directions were significant, indicated that resilience and stress partially mediated the relationship of mindfulness to happiness (Figure 1).

**TABLE 1:** Mean, Standard Deviations (SD), and intercorrelations among study measures.

Measure	Mean	SD	1	2	3
(1). Mindfulness	3.9	0.74			
(2). Resilience	2.6	0.61	0.27**		
(3). Happiness	4.5	1.2	0.31**	0.43**	
(4). Stress	1.1	0.51	−0.33**	−0.26**	−0.29**

**Correlation is significant at the 0.01 level (2-tailed).

Then, we tested Model 2, a full mediation model having direct path from mindfulness to happiness constrained to zero. The comparison of Model 1 and Model 2 was conducted using chi-square difference test. The fit of the Model 2 decreased significantly, after eliminating the above direct path from mindfulness to happiness, (Δχ2 (1, *N* = 523) = 10.13, *p* < 0.001). The comparison of results between Model 1 and Model 2 indicated that Model 1 was a better fit than Model 2. To find out the best model, alternative models were also tested. In Model 3 (first alternative model), we added a path from resilience to stress. Model 3 in comparison to Model 1 provided a better fit to the data: χ2 = 81.87; df = 59; RMSEA = 0.027; SRMR = 0.031; and CFI = 0.992. The chi-square difference was also found significant (Δχ2 (1, *N* = 523) = 18.69, *p* < 0.001). Based on the comparison between Model 1 and Model 3, we chose Model 3 as our final structural model (Figure 1).

The mediation effects of resilience and stress on the relationship of mindfulness and happiness were tested using bootstrapping procedures in AMOS. Using the original data set (*N* = 523), by random sampling, we generated 10,000 bootstrapping samples. The mediating effects of resilience and stress and their associated 95% confidence intervals are shown in Table 2. 95% CI values of indirect effects of mindfulness on happiness indicated that resilience and stress significantly mediated the relationship between mindfulness and happiness.

**TABLE 2:** Bootstrapping indirect effects and 95% confidence intervals (CI) for the mediational model.

Model Pathways	Point estimates	95% CI	
		Lower	Upper
Mindfulness→Resilience→Happiness	0.22	0.13	0.34
Mindfulness→Stress→Happiness	0.10	0.03	0.20
Resilience→Stress→Happiness	0.08	0.02	0.18
Mindfulness→Resilience→Stress	−0.043	−0.08	−0.02
Mindfulness→Resilience→Stress→Happiness	0.02	0.01	0.05

We further examined various alternative models to determine the best model. These alternative models were formed using different associations of the study variables. An alternative model (Model 4) was tested with mindfulness as mediating variable, resilience/stress as exogenous variables, and happiness as outcome variable. Model 4 also fit the data well: χ2 = 121.83, df = 60; RMSEA = 0.044; PClose = 0.781; SRMR = 0.078; and CFI = 0.979. Another alternative model (Model 5) was tested with happiness as exogenous variables, mindfulness as outcome variable, and resilience/stress as mediators. This model also fit the data well: χ2 = 93.88, df = 60; RMSEA = 0.033; PClose = 0.990; SRMR = 0.042; and CFI = 0.988. However, from the comparison of the results of the above five models, we found that Model 3 was the best fit. As shown in Table 3, Model 3 had smaller AIC and ECVI values than the other models. Model 3 also had the better fit to data. Thus, in conclusion, the preferred model (Model 3) indicated that resilience and stress partially mediated the relationship between mindfulness and happiness.

**TABLE 3:** Fit indices among competing models.

	χ2	df	χ2/df	RMSEA	SRMR	CFI	AIC	ECVI
Model 1	100.56	60	1.68	0.036	0.051	0.986	162.56	0.311
Model 2	110.69	61	1.82	0.040	0.055	0.983	170.69	0.327
Model 3	81.87	59	1.39	0.027	0.031	0.992	145.87	0.279
Model 4	121.83	60	2.03	0.044	0.078	0.979	183.83	0.352
Model 5	93.88	60	1.57	0.033	0.042	0.988	155.89	0.299

*N = 523, RMSEA = root mean square error of approximation; SRMR = standardized root-mean-square residual; CFI = comparative fit index; AIC = Akaike information criterion; and ECVI = expected cross-validation index.*

In the first regression analysis, the dependent variable was happiness. Age and gender were entered at Step 1. These two variables accounted for small and insignificant variance in happiness (R^2^ = 0.004). The mindfulness score was entered at Step 2 and was a significant predictor of happiness, accounting for an additional 8.8% of the variance and raising R^2^ to 0.092. The resilience score was entered at Step 3 and was also a significant predictor, accounting for an additional 13.7% of the variance and increasing R^2^ to 0.229. At Step 4, the interaction of mindfulness and resilience was a significant predictor of happiness accounting for an additional 1.5% of the variance and raising R^2^ to 0.244. Thus, findings support moderation by resilience of the relationship between mindfulness and happiness.

### Gender Differences

In order to examine gender differences. We compared the first model (which allows the structural paths to vary across sexes) with the second model (which constrains the structural paths between males and females to be equal). We tested the invariance in factor loadings between the two groups, i.e., male and female, and found no significant difference between the first model and the constrained model, Δχ2 (6, *N* = 523) = 4.73, *p* = 0.579. This suggested that there were no significant gender differences. We also tested the path coefficients for each of the relationships and found that all paths didn’t differ across sexes.

### Age Differences

We divided the sample into two groups of 17-20 years and 21-24 years, respectively. We tested the invariance in factor loadings between the two groups, and found no significant difference between the first model and the constrained model, Δχ2 (6, *N* = 523) = 1.34, *p* = 0.970. This suggested that there were no significant age differences. Path coefficients for each of the relationships were also tested, and it was found that all paths didn’t differ across the two ages.

## Discussion

The current study examined the relationship of mindfulness and happiness along with the mediation effects of resilience and stress on this relationship. To our knowledge, this is the first study to explore two perceived opposite mediators together of the relationship of mindfulness and happiness. Results of the study show that mindfulness is positively related to resilience and happiness; and is negatively related to stress. Results also show that resilience is positively related to happiness, negatively related to stress, and moderates the relationship between mindfulness and happiness thus strengthening its role in inculcating happiness. These results are consistent with earlier studies showing positive relationships between mindfulness and happiness (Coo and Salanova, 2018); mindfulness and resilience (Pidgeon et al., 2014; Hwang et al., 2018); and resilience and happiness (Lü et al., 2014). The results are also in line with prior research that indicates negative relationship between mindfulness and stress (Palmer and Rodger, 2009; Tran et al., 2014); resilience and stress (Hao et al., 2015); and stress and happiness (Abdollahi et al., 2014).

Furthermore, the findings of the current study reveal that resilience and stress partially mediate the relationship of mindfulness to happiness. These findings imply that resilience and stress might explain the relationship between mindfulness and happiness, to an extent. It means individuals with high levels of mindfulness are likely to show enhanced resilience, which in turn contributes to higher levels of happiness. Individuals with higher mindfulness levels are also likely to show lower stress, which in turn contributes to higher levels of happiness. A potential interpretation may be that mindful individuals have higher regulation of attention, emotion (Chambers et al., 2009), and higher self-control (Black et al., 2011). These abilities are the backbone of developing better resilience (Wills and Bantum, 2012; Jain and Cohen, 2013), and since resilience in the face of stress is a key aspect of a healthy brain (McEwen, 2016) as it allows better coping up with stress, it subsequently increases happiness (Feder et al., 2009; Fletcher and Sarkar, 2012; Lü et al., 2014). In addition, mindfulness is related with increase in self-regulation (Hülsheger et al., 2013), and decrease in stress and negative emotions (Jimenez et al., 2010). Therefore, overall mindfulness may lead to increase in resilience and decrease in stress, which on their turn increase happiness.

From a theoretical perspective, our study strengthens the postulation of mindfulness in self-determination theory. In line with existing proposition that mindfulness helps to bring about autonomous form of motivation (Ryan et al., 2021), our study gives evidence that students with higher mindfulness tend to be more aware of their internal phenomena such as needs and emotions and external phenomena such as conflicts and pressure, and are less likely to get influenced by automatic responses (de Bruin et al., 2015). As such, they are at a better position to engage in choices that are reflective and actions that are congruent with their self (Donald et al., 2020).

Our study also extends the literature on mindfulness as it suggests a pathway of the positive effects of mindfulness on happiness through increasing resilience and simultaneously lowering stress. Most of the early research has focused on either reducing stress or enhancing resilience. In contrast, our research is the first study, to the best of our knowledge that examines the combined role of increasing resilience and lowering stress to enhance happiness. Specifically, our research suggests that higher levels of mindfulness inculcate high levels of resilience, which helps in lowering stress and as result, increasing happiness. Our findings thus replicate and extend previous findings linking mindfulness to happiness among university students (Palmer and Rodger, 2009; Miller et al., 2017; Galante et al., 2018) and offer a better explanation of the process by which this occurs. Interestingly, whereas much of the research has focused separately on how mindfulness leads to lower stress and increase resilience, our study shows a dual effect of both stress and resilience on happiness.

Our study conveys several important implications for student development at university level. As mindfulness is negatively related to stress, it may have the potential to reduce their difficulties (Kabat-Zinn, 1990) and enhance their psychological well-being (Baer, 2003). The findings strengthen the adaptive qualities of mindfulness for students who may use it to cope with stress at the time of transitioning to the university. Adolescent phase particularly puts a lot of demand on the students at the time of adjusting in the university environment leading to high levels of stress. As the university students face high levels of stress, it is important to devise interventions to decrease stress and enhance well-being (Ward-Griffin et al., 2018).

From a practical perspective, the study findings can help in designing interventions that help individuals increase their resilience and decrease their stress in order to make a positive impact on mental health. Psychosocial factors that are associated with resilience include high coping self-efficacy, ability to reframe adversity in a more positive light, attention to health, good cardiovascular fitness, and the capacity to quickly recover from stress (Southwick and Charney, 2012). These resilience-promoting psychosocial factors may be cultivated through mindfulness training, which can be beneficial for an individual throughout the life span. Thus, the current study may be beneficial in giving suggestions for designing appropriate interventions for enhancing happiness by increasing resilience, lowering stress.

## Limitations and Future Directions

The current research has some limitations that should be addressed. First, a cross-sectional research design has been used, making it difficult to draw any causal relationships among the study variables. In order to overcome this limitation, longitudinal or experimental designs may be used in future research. The second limitation is the use of self-report measures in assessing the variables, which poses a threat to internal validity as respondents may be biased, due to social desirability. To reduce self-report bias, multiple methods (e.g., peers, parents, and friends) for evaluation can be used in future research. Third limitation is the use of a student sample. We encourage future research to expand this by including other samples such as working professionals. Fourth limitation is that no information was collected regarding the presence of any mental disorder in the sample of students. We suggest using multi-facets mindfulness scales, and to explore which facets of mindfulness relate most to resilience, happiness, and stress. The role of other mediating variables such as psychological capital, altruism, and psychological flexibility may be also tested to further understand the mindfulness-happiness relationship. We believe it will be important to further explore the effects of social support, emotional intelligence, and parents’ mindfulness on happiness through resilience and stress.

## Data Availability Statement

The raw data supporting the conclusions of this article will be made available by the authors, without undue reservation.

## Ethics Statement

Ethical review and approval was not required for the study on human participants in accordance with the local legislation and institutional requirements. The patients/participants provided their written informed consent to participate in this study.

## Author Contributions

All authors listed have made a substantial, direct, and intellectual contribution to the work, and approved it for publication.

## Conflict of Interest

The authors declare that the research was conducted in the absence of any commercial or financial relationships that could be construed as a potential conflict of interest.

## Publisher’s Note

All claims expressed in this article are solely those of the authors and do not necessarily represent those of their affiliated organizations, or those of the publisher, the editors and the reviewers. Any product that may be evaluated in this article, or claim that may be made by its manufacturer, is not guaranteed or endorsed by the publisher.
